# Vimentin: from a cytoskeletal protein to a critical modulator of immune response and a target for infection

**DOI:** 10.3389/fimmu.2023.1224352

**Published:** 2023-07-05

**Authors:** Jeffrey Arrindell, Benoit Desnues

**Affiliations:** ^1^ Aix Marseille Univ, Institut de Recherche pour le Développement (IRD), Assistance Publique-Hôpitaux de Marseille (AP-HM), Microbes Evolution Phylogeny and Infections (MEPHI), Marseille, France; ^2^ Institut Hospitalo-Universitaire (IHU)-Méditerranée Infection, Marseille, France

**Keywords:** vimentin, intermediate filaments, immune response, viral infections, bacterial infections

## Abstract

Vimentin is an intermediate filament protein that plays a role in cell processes, including cell migration, cell shape and plasticity, or organelle anchorage. However, studies from over the last quarter-century revealed that vimentin can be expressed at the cell surface and even secreted and that its implications in cell physiology largely exceed structural and cytoskeletal functions. Consequently, vimentin contributes to several pathophysiological conditions such as cancer, autoimmune and inflammatory diseases, or infection. In this review, we aimed at covering these various roles and highlighting vimentin implications in the immune response. We also provide an overview of how some microbes including bacteria and viruses have acquired the ability to circumvent vimentin functions in order to interfere with host responses and promote their uptake, persistence, and egress from host cells. Lastly, we discuss the therapeutic approaches associated with vimentin targeting, leading to several beneficial effects such as preventing infection, limiting inflammatory responses, or the progression of cancerous events.

## 1 Introduction

Intermediate filaments are composed of approximately 70 different types of proteins such as keratin, vimentin, desmin, and lamin. The proteins that compose the intermediate filament depend on the cell type and its localization. Intermediate filaments are approximately 8-12 nm wide; they are called intermediate because they are in between the size of microfilaments and microtubules (1). Besides lamins, which are found in the nucleus and help support the nuclear envelope (2), intermediate filaments are mainly found in the cytoplasm although nestin and vimentin can be found in the nucleus (3, 4). In the cytoplasm, intermediate filaments maintain the cell shape and tension and provide structural support to the cell.

Vimentin is expressed in mesenchymal cells, including fibroblasts, endothelial cells, macrophages, melanocytes, Schwann cells, and lymphocytes (5). It is known to be implicated in a dynamic, flexible network that plays an important role in several cell events. Most of the knowledge regarding the role of vimentin comes from studies on vimentin-deficient mice. While the phenotype of such mice is rather mild (6), detailed analyses have shown that vimentin deficiency affects cell adhesion, migration, and cell signaling and, therefore, that vimentin plays key roles in several physiological processes (5).

One of the processes that implicate vimentin is the epithelial-to-mesenchymal transition (7), which allows polarized cells to revert to a mesenchymal phenotype, granting the cells a greater migratory ability and a more resistant cell type (8). Vimentin also regulates cell adhesion by interacting with and regulating integrin function (9). In addition, as a major intermediate filament protein in leukocytes, vimentin plays a critical role in leukocyte migration by regulating cell attachment to vascular endothelium and transmigration (10).

However, while vimentin is a cytoskeletal protein, several reports have shown that in macrophages and microvascular endothelial cells, it can be expressed at the cell surface or secreted, suggesting a role during innate immune responses (11–14). Thus, it is not surprising that some pathogens have evolved strategies in order to subvert vimentin function and interfere with host responses.

In this review, we will highlight the different roles that vimentin can play during pathogen infection of the host cell.

## 2 Vimentin structure

**Figure 1:**
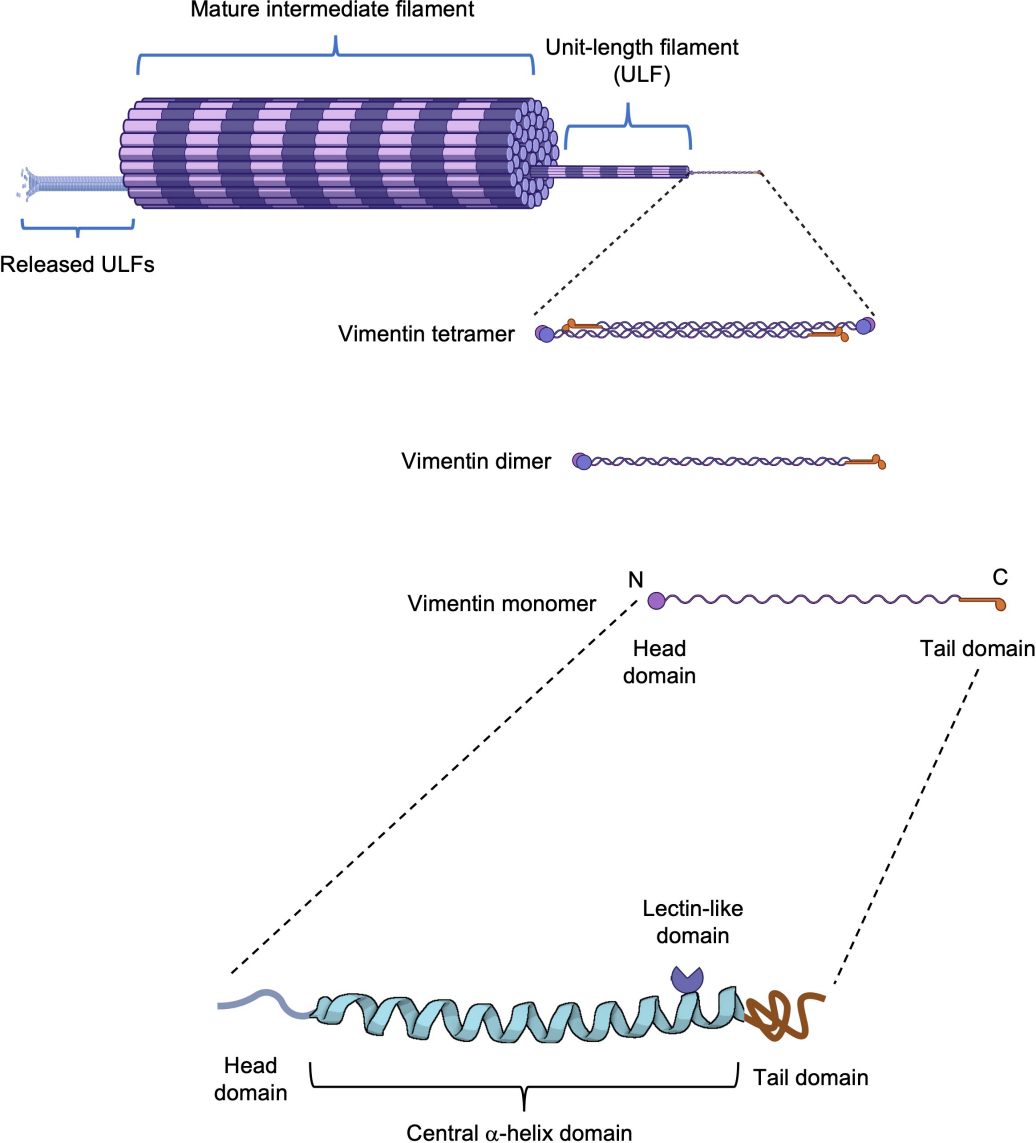
Vimentin structure. Schematic representation of a vimentin filament from a monomer to its assembly into a mature filament. The vimentin monomer is composed of a central α-helical domain flanked by an unstructured head and tail domain. On the central domain, vimentin possesses a rod II domain with lectin-like properties.

The basic structure of vimentin consists of a central α-helical rod domain flanked by unstructured head and tail domains. Vimentin monomers pair up into coiled-coil dimers, which then align in a staggered, antiparallel fashion to form tetramers; groups of eight tetramers make up the unit-length filaments (ULFs) that join end-to-end and subsequently undergo radial compaction to form the mature vimentin intermediate filaments (15). Interactions between vimentin molecules are regulated by posttranslational modifications, including O-linked glycosylation and phosphorylation (16). O-GlcNAcylation of Ser49 residue (and Ser34, Ser 39 to a lesser extent) in the head domain promotes interactions between vimentin molecules and the assembly and/or maintenance of mature vimentin filaments (17). The formation of vimentin filaments is dynamic although ULFs are released from growing filaments at low rates. The equilibrium between the abundance of free ULFs and assembled filaments favors polymerization, leading to the formation of a dynamic, complex, and insoluble network of filaments (**Figure 1**) that plays an important role in several cellular events. Disassembly and assembly of vimentin filaments are mainly orchestrated by serine phosphorylation (18, 19), which regulates severing and annealing events (20, 21). Inhibition of type-1 and type-2A protein phosphatases (PP1 and PP2A) results in the disassembly of vimentin filaments into soluble vimentin tetramers (19), whereas several serine/threonine kinases, including protein kinase A and C (PKA and PKC) (19), calmodulin-dependent protein kinase II (CaMKII) and Rho-associated protein kinases (ROCK1 and ROCK2) (22–24), have been involved in vimentin phosphorylation and linked to diverse biological processes. In addition, it has been shown that tyrosine phosphorylation and dephosphorylation by Src and SHP2, respectively, are also involved in the reorganization of vimentin filaments during migration in response to growth factors (25).

During mitosis, vimentin filaments are dramatically reorganized and may appear as a filament cage around the mitotic spindle or may disassemble, depending on the cell type. Vimentin disassembly is mediated by phosphorylation by various kinases, including Cdk1 (maturation/M-phase promoting factor, MPF; p34^cdc2^/cyclin B) and Plk1 from prometaphase to metaphase (26–28) and by Aurora-B and Rho-kinase from anaphase to the end of mitosis (24, 29). Interestingly, vimentin disassembly or persistence as a filament cage appears dependent on nestin, an intermediate filament protein that cannot form filament by itself but that can form copolymers with vimentin (30), thereby promoting its phosphorylation and disassembly (31). In nestin-negative cells, vimentin persists as filaments that closely interact with the actomyosin cortex and redistribute to the cell periphery (32).

### 2.1 Vimentin and organelle anchorage

Vimentin forms a vast intracellular network surrounding the nucleus and spanning toward the cell periphery. The vimentin distribution throughout the cell allows the structural maintenance of the cell organelles.

It has been previously shown that vimentin is able to interact with different organelles such as the Golgi apparatus, mitochondria, or even vacuoles via associated proteins.

The formimidoyltransferase cyclodeaminase (FTCD), an enzyme located on the Golgi membrane plays the role of a linker protein, promoting the binding of vimentin to the Golgi apparatus as revealed by the colocalization of vimentin with GM130, a marker protein of the Golgi apparatus (33). The interaction between FTCD and vimentin allows the remodeling of the Golgi and represents an interaction interface between the Golgi and the rest of the cytoskeleton (33). In addition, it was recently shown that the trans-Golgi network coiled-coil protein GORAB interacts with vimentin (34), suggesting that vimentin filaments contribute to the structural stability of the Golgi apparatus through GORAB, although it cannot be excluded that vimentin binds Golgi via other proteins.Vimentin has also been shown to be associated with proteins implicated in the sorting of the endosomal-lysosomal machinery (35). One of the key proteins implicated in the sorting of the proteins present in distinct vesicles secreted by host membranes is the adaptor protein (AP) complex (36). More specifically the AP-3 complex implicated in the sorting of proteins present on lysosomes interacts directly with vimentin (35), suggesting the potential role that vimentin plays in the scaffolding of lysosome vesicles throughout the cell and also the regulation of the sorting ability of AP-3 complex (35).

Finally, vimentin is implicated in the cellular movement of the mitochondria. Plectin 1b, a cytolinker, ensures the anchorage of the mitochondria to vimentin filaments (37) but it also seems that vimentin can bind directly to the mitochondria via its N-terminal domain (38). The binding of vimentin filaments to the mitochondria modulates the motility of these organelles and limits the movements of the mitochondria throughout the cells.

### 2.2 Vimentin and cell shape and motility

Besides the roles that vimentin plays in organelle anchoring, it also confers the cell’s general rigidity and shape.

Polarized epithelial cells undergo epithelial-to-mesenchymal transition (EMT), which is a process that confers the cells the capacities of migration and to become more resistant. EMT often occurs during wound healing where the polarized epithelial cells detach from the base and migrate to the wound site, reverting to their initial state. Vimentin governs the healing process by regulating fibroblast proliferation, extracellular matrix (ECM) accumulation, and EMT processing (39). This process is mostly mediated by inflammatory signals and ECM proteins, including collagens, laminins, elastin, and tenacins (8). Cells undergoing EMT have increased vimentin expression (7), which confers the cell its elasticity to navigate to rigid domains. Vimentin also protects against compressive stress (40) and is involved in mechanosensing during migration by enhancing cell spreading (41).

It has been shown that migratory cells powered by EMT utilize vimentin to generate cell extension and direct migration. This process is controlled by vimentin phosphorylation by p21-activated kinase 1 (42) after interaction with the actin-binding protein filamin A (43, 44). The mature phosphorylated vimentin filaments are stable and maintain the cell extensions that are directed by the detection of the inflammatory signals or ECM complex (45).

Other cells such as lymphocytes also utilize the reorganization of vimentin to initiate their movements through a dense environment or during transmigration (10).

During cancer progression, epithelial cells can undergo EMT to migrate, invade, and proliferate and vimentin overexpression can be a sign of the progression of cancerous events (46), including breast cancer, prostate cancer, endometrial cancer, or gastrointestinal tract tumors. In the case of breast cancer, tumorigenic events, such as tumor cell migration and invasion of cancer cells, are highly correlated to the overexpression of vimentin (47, 48). The expression of vimentin in cancerous events can also serve as a prognostic marker, for example in gastric cancer where the overexpression of vimentin is linked to metastasis (49).

### 2.3 Extracellular and membrane-associated vimentin

Besides its role in maintaining the cell organelles, giving the cell its global shape and rigidity, and acting as a moving scaffold to direct the migration of the cells, several studies have shown that vimentin can be expressed at the cell membrane, secreted to the extracellular environment and even be secreted via exocytosis of particles.

Indeed, both vimentin and desmin, another type III intermediate filament protein, can be located at the cell surface of cardiomyocytes and vascular smooth muscle cells (50). Interestingly, the rod II domain (**Figure 1**) present on both vimentin and desmin has a carbohydrate-recognition activity that binds β-*N*-acetylglucosamine (GlcNAc). It was shown that this lectin-like activity was required for the internalization of GlcNAc-conjugated liposomes by cardiomyocytes, suggesting that cell surface vimentin and desmin are involved in the clearance of GlcNAc-conjugated proteins and cellular debris (50).

It was also shown that in certain conditions, including infection, immune cells such as monocytes or macrophages can express vimentin at the membrane (12, 51) or even secrete vimentin (11), suggesting a role during the immune response. Interestingly, the secretion of vimentin is triggered by the pro-inflammatory cytokine TNF produced by macrophages, whereas it is inhibited by the inhibitory cytokine IL-10 (11). Finally, astrocytes, neutrophils, and endothelial cells have also been shown to secrete vimentin (52–55). Likewise, tumor endothelial cells overexpress and secrete vimentin through type III unconventional secretion mechanisms (56), although cancer cells can also secrete vimentin via the use of exosomes (57). As mentioned above, tumorous cells overexpress vimentin and the presence of vimentin in exosomes is most likely due to the overexpression of vimentin and this might likely accelerate the transformation of the target cells into an uncontrollable EMT (46).

Vimentin is also involved in several inflammatory and autoimmune diseases including, among others, rheumatoid arthritis, systemic lupus erythematosus, sarcoidosis, or ankylosing spondylarthritis (58). Vimentin is a substrate of peptidylarginine deiminase type 2 (PADI2), which deiminates arginine residues into citrulline residues, resulting in a mutated citrullinated vimentin (59). Vimentin citrullination results in a loss of vimentin’s normal functions but also triggers the production of anti-citrullinated protein antibodies (ACPAs) (60), which have been shown to induce osteoclastogenesis and bone loss (61, 62). As a consequence, anti-citrullinated antibodies and more generally ACPAs represent a valuable diagnostic and prognostic marker of rheumatoid arthritis (63, 64).

## 3 Vimentin and immune response

To detect and neutralize pathogens, the host cells mobilize various mechanisms. One of the mechanisms is the use of sensor proteins or pattern recognition receptors that recognize conserved motifs expressed by microbes and that include Toll-like receptors (TLRs) (65), C-type lectin receptors (CLRs) (66), Nucleotide-binding and oligomerization domain (NOD)-like receptors (NLRs) (67). One of the major responses from these signaling proteins is the alteration of gene expression through the activation of the nuclear factor-κB (NF-κB), mitogen-activated protein kinase (MAPK), or interferon (IFN)-regulatory factor (IRF) pathways. The genes affected by these alterations in transcription are mostly those encoding pro-inflammatory cytokines and IFN-stimulated genes and they play a major role in cell-intrinsic control of pathogens and activation of adaptive immunity. NLRs detect bacterial infection and assemble signaling structures called inflammasomes (67), large oligomeric multiprotein complexes that act as signaling platforms to catalytically activate pro-caspase 1 to promote the maturation of IL-1β and IL-18 pro-inflammatory cytokines (68).

**Figure 2:**
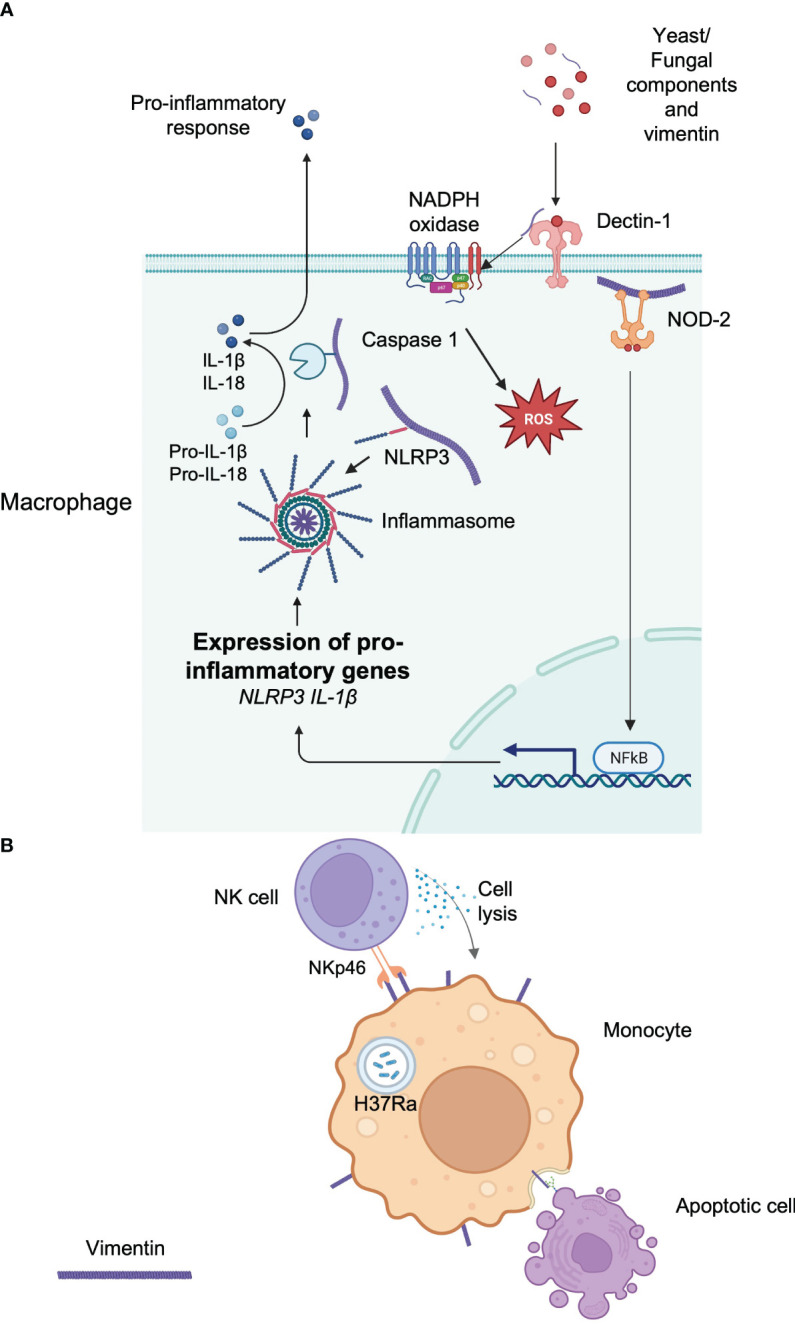
Implication of vimentin in the immune response. **(A)** Intracellular vimentin implications of the initiation or facilitation of an immune response to a signal. Extracellular vimentin is also detected by Dectin-1, which initiates the production of intracellular ROS. The vimentin network plays an important role in anchoring or transporting essential proteins implicated in the immune response. **(B)** Extracellular vimentin is implicated in the recognition of apoptotic cells via its lectin-like domain. The response of an infection by a pathogen can increase extracellular vimentin, which can be detected by circulating immune cells.

Several studies have revealed that the vimentin network plays an essential role in the detection of pathogens as well as in the mobilization of antimicrobial responses. Indeed, it has been demonstrated that recognition of muramyl-dipeptide (MDP), the minimal motif of bacterial peptidoglycan by the intracytoplasmic sensor NOD2, required membrane targeting of NOD2 for proper activation of NF-κB after MDP recognition (69). Unexpectedly, vimentin is directly implicated in this process (**Figure 2A**). NOD2 interacts with vimentin at the plasma membrane through its leucine-rich repeats domain and this is critical for NF-κB activation since vimentin inhibition with Withaferin A relocalizes NOD2 in the cytosol and inhibits downstream activation of NF-κB as well as NOD2-dependent autophagy induction (69). Interestingly, in light of *card15*, which encodes the NOD2 protein as a susceptibility gene for Crohn’s disease, polymorphisms in the *vim* gene may also be associated with Crohn’s disease, although further genetic studies are required (69).

Vimentin also regulates the NLRP3 inflammasome. Vimentin-deficient mice are protected against acute lung injury and fibrosis (70). This is because vimentin binds NLRP3 in macrophages and this interaction may facilitate the transport and the assembly of other proteins implicated in the inflammasome, including caspase-1, which is implicated in the maturation of IL-1β (70) (**Figure 2A**).

Finally, it has been shown that vimentin could be an endogenous ligand for the CLR Dectin-1 (71) (**Figure 2A**) and mediate chronic inflammation leading to atherosclerosis. Although Dectin-1 is implicated in the recognition of yeast and fungal pathogens (72), it can also bind extracellular vimentin that is released during necrosis in atherosclerotic lesions, leading to NADPH oxidase activation and ${\text{O}}_{2}^{-}$ production and low-density lipoprotein oxidation, thereby contributing to the chronicity of the disease (71). This is in agreement with other findings that showed that vimentin deficiency attenuates atherosclerosis in mice (73). However, the precise role of vimentin in inflammation remains to be further clarified since it was also shown that macrophages deficient in vimentin are characterized by increased oxidative and inflammatory responses (73).

Vimentin was shown to regulate host antiviral immune responses. Indeed, *in vitro*, vimentin controls type-I IFN expression induced by TLR, retinoic acid-inducible gene-I (RIG-I)-like receptors (RLR), and the cytosolic DNA sensor cGAS by directly interacting through its N terminus domain with both TBK1 and IKKε and thus preventing IRF3 phosphorylation (74). These data are strengthened by the fact that mice deficient in vimentin are more resistant and display milder symptoms following infection by the encephalomyocarditis virus or herpes simplex virus (HSV-1) (74).

Besides its involvement in various signaling pathways that controls host responses, vimentin can also participate directly in the immune response by acting as a ligand when expressed at the cell surface. For example, vimentin present at the surface of infected monocytes is used as a recognition pattern for Natural killer (NK) cells (12). NK cells naturally utilize the activating receptors NKp30 and NKp46 for the recognition and lysis of cells expressing their ligands to initiate their lysis (75). Interestingly, monocytes infected by *M. tuberculosis* (H37Ra) upregulate vimentin at the cell surface and NK cells are able to detect infected cells by interacting with vimentin through NKp46, which in turn induces the lysis of the infected cells (12) (**Figure 2B**). In addition, surface expression of vimentin may also participate in the engulfment and clearance of apoptotic cells by interacting with O-GlcNAc proteins present in apoptotic cells (76) (**Figure 2B**). Finally, as already noted above, activated macrophages secrete vimentin, and extracellular cell-surface vimentin is mainly localized at the rear extremity of the macrophage, in the opposite direction of the migration (51).

## 4 Diversion of vimentin by pathogens

Given the multiple roles that vimentin plays as a structural entity maintaining the overall cell integrity, its role in the regulation of the immune response, and its availability at the cell surface in certain conditions, it is not surprising that certain pathogens make use of vimentin to facilitate their entry into the host cell or allow their maintenance or to disseminate to different organs (77, 78).

**Figure 3:**
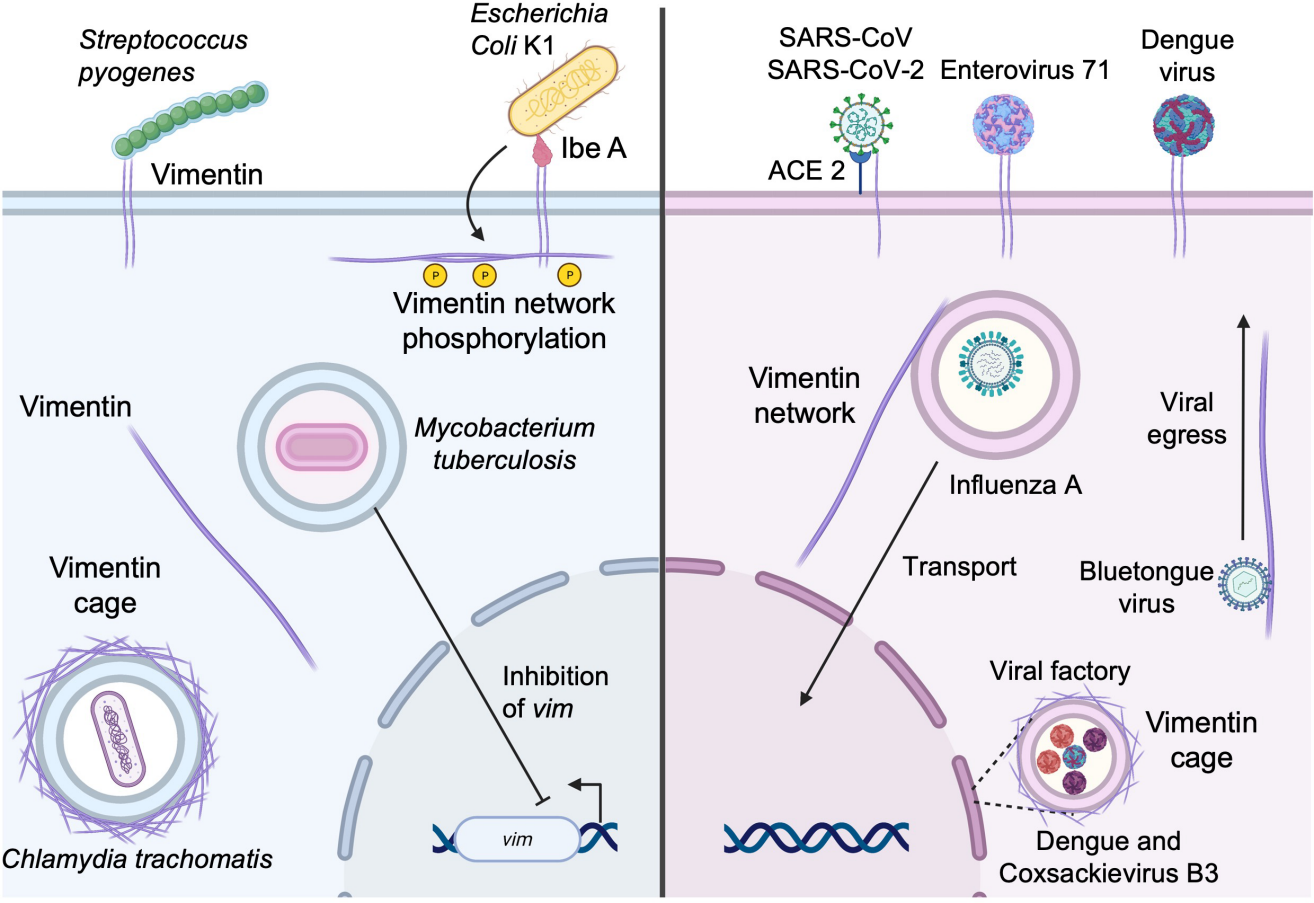
Subversion of vimentin by bacterial and viral pathogens. Vimentin expressed at the cell surface can be utilized by pathogens as an attachment factor to initiate entry into the host cell. Vimentin can also be recruited for the establishment and maintenance of replicative niches and the vimentin network may also facilitate the egress of newly formed viral particles (see text for details).

*Escherichia coli* is a common Gram-negative bacterium found in the gastrointestinal tract, which is in most cases harmless. However, strains expressing virulence factors such as Ibe proteins (IbeA, IbeB, IbeC, IbeR, and IbeT) can cause neonatal bacterial sepsis and meningitis (NSM) in immunocompromised infants, with high morbidity and mortality (79). Most cases of NSM are caused by *E. coli* K1 (79), which expresses IbeA. IbeA is required for invasion of human brain microvascular endothelial cells (HBMEC) where it interacts with surface-expressed vimentin (**Figure 3**) at the head domain (80). IbeA-vimentin interaction then induces a signaling cascade involving the phosphorylation of vimentin and also the activation of the MAPK signaling pathway that mediates the invasion of *E. coli* K1 into HBMECs and the activation of NF-κB, which are required for bacteria-mediated pathogenicity (81, 82).

Other bacteria utilize cell surface vimentin as a means of entry. *Streptococcus pyogenes* (group A *streptococcus*, GAS) is a Gram-positive coccus, member of the skin microbiota. Under certain circumstances, it may be responsible for several human diseases ranging from mild skin infections and pharyngitis to necrotizing fasciitis and myonecrosis (83). In patients lacking a portal of entry, GAS is suspected to spread from the oropharynx to the site of prior muscle injury. There, GAS binds vimentin that is overexpressed by injured muscle cells. Adhesion of GAS at the cell surface (**Figure 3**) in sufficient numbers seems to lead to the initiation of infection of the host cell (83). In addition, *S. pyogenes* expresses SyaA, an ADP-ribosyltransferase that modifies vimentin at the head domain (84). Ribosylation of vimentin at the head domain leads to the disruption of the vimentin network around the nucleus, and this disruption may interfere with wound healing, play a role in reducing the microbicidal activity of macrophages, and contribute to further bacterial dissemination.

Once inside the cell, some intracellular bacteria, including *Chlamydia trachomatis*, use vimentin for maintaining the replicative vacuole. *C. trachomatis* are obligated intracellular Gram-negative bacteria that mainly infect ocular and genital epithelia leading to conjunctivitis, salpingitis, and urethritis (85). Vimentin has been shown to contribute to the establishment of replicative niches (**Figure 3**). Indeed, *C. trachomatis* maintains a large, stable membrane-bound vacuole that is stabilized by co-opting the function of F-actin and vimentin. The bacterium mobilizes the GTPase RhoA functions to assemble an actin ring around the vacuole. In addition to F-actin rings, a cage of intermediate filaments, including vimentin, is assembled on the mature chlamydial inclusion (86) and vimentin is necessary for the inclusion expansions because it provides scaffolding support. CPAF, a protease synthesized by *C. trachomatis*, can cleave the head domain of vimentin, allowing the expansion of the inclusion (86). Of note, the ability of vimentin to form cages may be reminiscent of the ring like-structures that form in the initial steps of cell adhesion/spreading as well as during mitosis (87).

*M. tuberculosis* is another pathogen that targets vimentin to favor infection. *M. tuberculosis* is a facultative intracellular bacterium that infects macrophages and monocytes and persists in vacuoles. *M. tuberculosis* downregulates both the reactive oxygen species (ROS) production and the pro-inflammatory response (88) to allow its persistence in the host cell. Interestingly, during *M. tuberculosis* infection, vimentin is also downregulated (**Figure 3**) and evidence suggests that the downregulation of vimentin is associated with that of ROS and depends on ESAT6 expression by *M. tuberculosis* (89).

Numerous studies have also shown that some viruses can use vimentin as a receptor/coreceptor, including dengue virus (90), enterovirus 71 (91), influenza A (92), severe acute respiratory syndrome coronavirus (SARS-CoV) (93), and SARS-CoV-2 (94–97) (**Figure 3**). Regarding betacoronaviruses, it was first determined that vimentin acted as a co-receptor alongside angiotensin-converting enzyme 2 (ACE2) on epithelial cells during the binding of the spike protein of SARS-CoV (93), which in turn allowed the entry of SARS-CoV into the host cell. It has been shown that the recently emerged SARS-CoV-2, highly similar to SARS-CoV (98), is also able to utilize vimentin at the cell surface as an attachment receptor (**Figure 3**) to facilitate the binding to ACE2. We have previously shown that inhibition of vimentin during SARS-CoV-2 infection reduced viral uptake, favored the protection against virus-mediated cell cytotoxicity, and reduced the pro-inflammatory response (95).

Influenza A virus, the causative agent of seasonal flu epidemics, reorganizes the vimentin network after infection of the host cell (99) (**Figure 3**). A recent study demonstrated that vimentin-deficient cells showed a massive decrease in the production of viral RNA and viral protein production (92). This was due to the lack of transportation of the endosomal vesicles containing the viral genomes to the nucleus, which is mediated by the vimentin scaffold throughout the cell (92).

Finally, during viral infection, vimentin can also form vimentin cages that facilitate viral replication and protein production of viruses. For example, dengue virus (DENV) and coxsackievirus B3 utilize vimentin to concentrate their viral factories to the perinuclear area (**Figure 3**), whereas, in vimentin-deficient cells, the viral factories are dispersed throughout the cell (100, 101). Similar findings were observed during Zika virus (ZIKV) infection (102). However, besides its structural role in maintaining the integrity of ZIKV replication complexes, vimentin additionally interacts with and regulates RNA-binding proteins to facilitate viral replication (102). Vimentin is also required for viral replication of the alphacoronavirus transmissible gastroenteritis virus (TGEV). Although the precise implication of vimentin needs to be further explored, it has been shown that vimentin interacts with the nucleocapsid N protein and TEGV replication is severely impaired in vimentin-deficient cells (103).

Vimentin may also be required for viral egress. This is the case for infection with bluetongue virus, whose capsid viral protein 2 (VP2) binds directly to cytosolic vimentin (104). The neo-formed mature viral particles of bluetongue virus utilize the interaction between vimentin and VP2 to migrate to the surface and allow its egress (104) (**Figure 3**). In another case, DENV-2 non-structural protein 1 (NS1), which is an indicator of viral replication, interacts with vimentin, and the disruption of vimentin results in a decrease of NS1 expression and decreased viral replication and viral egress (105). Lastly, the foot and mouth disease virus non-structural protein 2C interacts directly with vimentin, which forms a cage around viral factories (106). The interaction with vimentin and non-structural protein 2C seems to be implicated in viral replication and viral release since non-structural protein 2C mutant-expressing viruses have decreased viral replication and release (106).

## 5 Vimentin targeting as a therapeutic option

As mentioned above, vimentin is involved in numerous physiological and pathophysiological processes. Hence, given the multiple localizations of vimentin, several therapeutic strategies have been developed to target vimentin in light of tumorigenesis and viral and bacterial infections.

Several studies have shown that cell surface vimentin can be detected and potentially blocked by using monoclonal antibodies directed against vimentin (107–110). The overexpression of vimentin in most cases is a marker of EMT, and uncontrolled EMT leads to the initiation of cancerous cell establishment. Hence, targeting extracellular vimentin with the monoclonal 86C antibody induces the apoptosis of glioblastoma multiforme cancer stem cells (109). In addition, the monoclonal SC5 antibody has been used to identify target cells in the case of the cutaneous T cell lymphoma Sézary syndrome (107). Pritumumab is another anti-vimentin monoclonal antibody used in glioma patients that binds to malignant cells expressing vimentin at their surface and is able to distinguish physiological and malignant vimentin (108, 110). Besides monoclonal antibodies, a polyclonal response induced by vaccination against extracellular vimentin seems very promising as it appears effective and safe in two different syngeneic preclinical mouse models of melanoma and colorectal carcinoma, as well as in dogs with spontaneous transitional cell carcinoma of the bladder (56). Cell surface vimentin has also been shown to be increased after an infection of the host cell by SARS-CoV-2 (95). This increase probably facilitates viral entry and targeting vimentin with the vimentin-targeting small molecule ALD-R491 (111, 112) seems to decrease viral entry (113). The subsequent decrease in entry is also associated with an increase in host cell survival and an alteration of the host response to the infection, which is still not completely understood (95). Thus, targeting extracellular vimentin seems to be an effective way to target specific cells undergoing abnormal modifications or to decrease viral uptake.

Intracellular vimentin could also be targeted as a therapeutic option by reorganizing its availability throughout the cell. Cytoplasmic vimentin is used by various pathogens (86, 106, 114) besides those cited above that can establish their niches or allow easier recruitment of host effectors to favor the pathogen cycle. Therefore, disruption of intracellular vimentin seems to be an effective way to limit the establishment or replication of pathogens within the host cell.

To target intracellular vimentin, several molecules [which have been reviewed extensively by Ramos et al. (115)] have been shown to have a disrupting effect on vimentin. One of the molecules is Withaferin A (WFA) (116), a natural compound extracted from *Withania somnifera*. WFA can bind directly to vimentin, leading to a disruption of the vimentin network throughout the cell. Interestingly, WFA has also a potent anti-tumoral and anti-angiogenic effect (116). Similarly, the natural compound Ajoene, which is extracted from garlic cloves, can bind to vimentin and disrupt the intermediate filament network (117). The disruption of vimentin leads to a decrease in cell migration and in the case of cancer cells, this decrease in migration is associated with a reduction of invasion of cancerous cells, giving it an antimetastatic activity (117). Finally, simvastatin, which is primarily used to lower blood cholesterol levels, has also been shown to bind and reorganize vimentin to one side of the nucleus, which later induces the apoptosis of the cells (118), thereby contributing to neutralizing cancerous cells (118). Hence, inhibition of vimentin seems to have various beneficial effects. Therapeutic targeting of vimentin is promising in cases of vimentin hijacking by pathogenic mechanisms, including infection and tumorigenesis.

## 6 Conclusion

Initially considered a redundant, non-essential intermediate filament protein, vimentin has quickly been demonstrated to be a key player in a variety of cell features. Vimentin functions range from a scaffolding network anchoring cell organelles, which can rearrange to allow cells to migrate and adapt their flexibility, to a critical regulator of several immune processes. The rearrangement of vimentin can also relocate vimentin to the cell surface, where the newly available vimentin is exposed to external ligands such as pathogens or surrounding cells. Vimentin can also be found in the extracellular environment. Vimentin engagement can potentially modulate immune signaling pathways but also facilitate the entry of a pathogen. Some pathogens have the ability to hijack or remodel the vimentin network to favor their life cycle. Over the years vimentin has been determined as a suitable target for therapies where compounds are able to disrupt the vimentin network and in turn block the effects of either pathogens or tumorous events.

## Author contributions

JA searched the literature and developed the first draft under the supervision of BD. BD conceptualized and revised the manuscript. All authors listed have made a substantial, direct, and intellectual contribution to the work and approved it for publication.
